# Preparation of Antibacterial Nanosilver Solution Microcapsules and Their Impact on the Performance of Andoung Wood Surface Coating

**DOI:** 10.3390/polym15071722

**Published:** 2023-03-30

**Authors:** Pan Pan, Xiaoxing Yan

**Affiliations:** 1 Co-Innovation Center of Efficient Processing and Utilization of Forest Resources, Nanjing Forestry University, Nanjing 210037, China; 2 College of Furnishings and Industrial Design, Nanjing Forestry University, Nanjing 210037, China

**Keywords:** antibacterial property, HLB value of emulsifier, microcapsule, paint film performance

## Abstract

In this paper, nanosilver solution was used as an antibacterial agent to prepare antibacterial microcapsules. The mass ratio of the core material to the wall material (W_core_: W_wall_), the emulsifier’s hydrophilic–lipophilic balance (HLB) value, the mass ratio of ethanol to the emulsifier in solvent (W_core_: W_emulsion_), and the rotational speed (r/min) were used to develop the four-factor, three-level orthogonal experiment, which was meant to investigate the most significant factors and the optimum process preparation parameters impacting the coating rate and yield of microcapsules. It was used to make an antibacterial coating that was applied to the surface paint film of a glass substrate and andoung wood, and it was mixed to the water-based primer with a content of 4%. Analyses of the mechanical, optical, and bactericidal characteristics were conducted. The micromorphology of the nanosilver solution microcapsules is influenced by the emulsifier’s HLB value. The color difference of the antibacterial coating film decreased with increasing emulsifier HLB value; however, the coating film’s gloss remained largely suitable. Additionally, the coating film’s transparency and tensile strength both decreased. It had minimal impact on the paint film’s surface hardness, but the adhesion and tensile strength showed a noticeable downward trend. The surface of the paint film was rough. *Escherichia coli* and *Staphylococcus aureus* were resistant to the antibacterial characteristics of the water-based primer film when it was combined with antibacterial nanosilver solution microcapsules by 80.7% and 74.55%, respectively. The coating film’s antibacterial properties were applied to the surface of the andoung wood, which were 75.7% and 71.0%, respectively, and somewhat decreased. In order to successfully inhibit bacteria, the nanosilver solution microcapsules were added to waterborne coatings. This ensures both the outstanding performance of the coating film and the effectiveness of the antibacterial effect. It expands the application prospects of antibacterial microcapsules in coatings.

## 1. Introduction

For a long time, microorganisms may deposit on the material surface once they contact the material [1,2], gradually adhere to the material surface through the interaction between microorganisms and the material surface [3], and then further grow on the material surface. The cell wall of wood is mainly composed of cellulose, hemicellulose, and lignin. Therefore, wood is vulnerable to erosion by insects as well as microorganisms such as mold, discoloring fungi, and decaying fungi, which lead to mildew, discoloration, and decay [4,5,6]. It causes the wood to be soft, change color, and be easy to peel off. The heartwood of andoung (*Monopetalanthus* spp.) is light-brown to pinkish-brown, with irregular dark stripes. Andoung wood is a porous material. It is slightly light, soft, medium-strength, and easy to process. Andoung wood has good nail grip and sanding performance. It was selected as the experimental substrate due to its corrosion resistance and rapid drying. However, this material is prone to deformation and cracking. It is often attacked by microorganisms such as bacteria. As a result, it is crucial to continue applying an antibacterial treatment to wood materials’ surfaces. As a new functional material, antibacterial materials have made great progress in material development and application [7,8]. Antibacterial coatings are applied to the surfaces of protected or decorated objects through different processes and antibacterial agents to form an adhesive, firm, continuous and strong antibacterial coating [9,10,11].

The most critical factor is antibacterial agents. A material can be endowed with antibacterial properties by adding a small amount of them to it, which can kill bacteria, fungi, and other microorganisms. Silver antibacterial agents have a wide range of antibacterial actions, making them useful in a variety of industries, including food engineering, biomedical technology, coating surface treatment, and agricultural production [12,13,14,15]. The novel multilayer nanocomposite HAP/Ag/SSD TNC created by Ge et al. [16] has strong sustained antibacterial activity, high effectiveness, and long activity. Kiriyama et al. [17] prepared an antibacterial polymer containing silver organic composite antibacterial agent, which can be dispersed in self-curing acrylic resin coating with good compatibility and easy dispersion [18,19]. It can ensure no chemical reaction with other components. After film formation, it does not affect its physical and chemical properties and has low volatility. Maryan et al. [20] synthesized nanosilver-treated polyurethane fiber with good antibacterial properties. Nguyen’s team [21] found that silver-plated polyurethane foam can be used as an excellent antibacterial water filter. Dat et al. [22] found that Staphylococcus aureus could be eliminated within 4 h at the concentration of Ag/GO, which verified the potential of antibacterial nanocomposites [23,24]. Nanoparticles are uniformly distributed in the polymer matrix. Ten harmful microorganisms are strongly inhibited and killed by nanosilver, but the first step is to make sure that its particle size is small [25]. It is easy to agglomerate when it is added to a water-based coating, resulting in the degradation of coating performance. However, commercially available nanosilver solution possesses consistent physical and chemical properties, great temperature tolerance, and long-lasting antibacterial effects. It can be prepared by simply being added to a water-based coating to create an antibacterial coating, ensuring the original coating’s outstanding performance. At present, antibacterial coatings are mainly produced by the physical mixing method, and this method is mixed with inorganic antibacterial agents to produce coatings that are easy to precipitate [26]. The coating can effectively shorten the antibacterial time and is easy to change color [27]. Microcapsule technology is a kind of particle technology that uses natural or synthetic polymer materials to coat dispersed solid or liquid substances, or even gases, to form a semipermeable or sealed capsule membrane [28,29,30]. The antibacterial microcapsules prepared by coating antibacterial agents with microcapsule technology have excellent performance [31,32]. The key in the preparation of microcapsules is the hydrophilic–lipophilic equilibrium value (HLB) of emulsifiers. Hydrophilic and lipophilic equilibrium value is used to measure the relative strength between the polar group and nonpolar group in the emulsifier molecule [33,34]. The combination of emulsifiers plays an emulsification and dispersion role in the microcapsule, forming an interface protection between the wall material and the core material. The issue of agglomeration can be avoided in this method, and the antibacterial agent can be released successfully.

Although earlier research has confirmed the viability of antibacterial microcapsules [35], the optimum method for creating silver microcapsules has not been investigated, and antibacterial efficacy still has to be enhanced. In this paper, urea-formaldehyde resin (UF) is used to cover nanosilver solution to create antibacterial microcapsules. An orthogonal experiment is used to further refine the antibacterial microcapsule preparation process, and the highest-performance microcapsules are selected. A certain quantity of additives is added to a water-based paint to create the antibacterial coating, which is then applied to the surface of the andoung wood. The antibacterial coating is initially placed to the glass plate to check the antibacterial ability of the coating itself, since the glass plate makes it difficult for bacteria to grow [36]. The surface of the andoung wood may be successfully shielded from microbial assault and have its service life increased by an antibacterial coating made by encapsulating nanosilver solution in microcapsules.

## 2. Materials and Methods

### 2.1. Test Materials

Table 1 lists the supplies needed for the experiment. The primary ingredients of a water-based primer are water-based acrylic copolymer dispersion, a matting agent, an additive, and water, with an approximate 30% solid content. The nanosilver solution has a silver ion content of 25 ppm, 99.9% pure silver, has particles that are 5–7 nm in size, and has the pH value of 3–5. The size of the andoung wood is 50 mm × 100 mm × 0.5 mm. The surface burr is removed by polishing with 800# sander, and the wood surface is smooth and flat.

### 2.2. Experimental Method

#### 2.2.1. Preparation of UF @ Nanosilver Solution Microcapsules

According to the relevant literature of microcapsules [37,38], the factors affecting the preparation of microcapsules are determined to include the mass ratio of core material to wall material (W_core_: W_wall_), the hydrophilic–liquid equilibrium (HLB) value of emulsifier, the ratio of ethanol to emulsifier in solvent (W_core_: W_emulsion_), and rotational speed (r/min). In order to eliminate the most important variables and investigate the ideal preparation method, the yield and coating rate are employed as the experimental findings. The HLB value of Span-80 and Tween-80 mixed emulsifier is calculated by the equation and the values of 4.5 for Span-80 and 15 for Tween-80 and their mass fractions. The equation is

*H = P*_*S*_ × *4.3 + P*_*t*_ × *15* (1)

calculated as follows:

*P_S_* represents the mass fraction of Span-80 in the mixed emulsifier, and *P_t_* represents the mass fraction of Tween-80.

The preparation methods of microcapsules mainly include:

(1) Preparation of wall materials: The ratio of 7.0 g urea, 9.47 g formaldehyde, and a certain amount of deionized water is 1:1. The pH of the solution is 8~9 with triethanolamine. The temperature of the water bath was controlled at 70 °C. The cup mouth is sealed with a layer of fresh-keeping film to prevent the prepolymer from deteriorating in contact with the air until the solution is colorless and transparent for about 30 min. After the beaker is taken out, it is cooled naturally.

(2) Preparation of core material solution: Certain amounts of ethanol, Span-80, and Tween-80 are mixed to obtain an emulsifier with a concentration of about 2% according to the dosage. At a temperature of 60 °C and rotating speed of 600 r/min, 8.4 g of nanosilver solution is slowly added to the emulsifier solution by pipette, and the core material solution could be obtained after stirring for 1 h.

(3) Preparation of UF @ silver nanosolution microcapsules: At a certain rotational speed, the urea formaldehyde prepolymer is added to the core material lotion, and the pH value of the solution is adjusted to about 2.5–3.0 with 8% citric acid monohydrate solution. The whole reaction process is reacted in a water bath for 3 h. After being placed for 3 days, the solution is filtered by vacuum suction filter and washed repeatedly with distilled water. Finally, the microcapsule powder is obtained after drying in a 60 °C oven for 48 h. The arrangement of orthogonal experiment is shown in Table 2, and the quantities of experiment are shown in Table 3 and Table 4.

#### 2.2.2. Preparation of Antibacterial Coating Film

The microcapsules with seven different HLB values are combined with the water-based paint primer at a mass fraction of 4% in accordance with the findings of earlier study [31]. The total mass of microcapsules and primer is 4.0 g. The application method is two coats of primer. After the first coat, it needs to be polished with 800# abrasive paper before being coated with the second coat of primer. After mixing evenly, it is coated on glass plate and andoung wood, dried for 20 min at room temperature, and then transferred to the oven for drying for 30 min to make the paint film solidify.

### 2.3. Testing and Characterization

#### 2.3.1. Characterization of Microcapsules

(1) Coverage rate: The 1.0 g microcapsule (*M*_1_) is weighed. After it is fully ground, it is fully soaked with a certain amount of ethyl acetate for 72 h. After being washed and filtered by deionized water, the remaining wall material mass (*M*_2_) is weighed after drying. The calculation formula of coverage rate (*C*) is as follows.

*C* = (*M*_1_ − *M*_2_)/*M*_1_ × 100% (2)

Output: The microcapsule powder is directly weighed and counted after drying.

(2) Microstructure analysis: Zeiss Axio Scope A1 optical microscope (OM) (Carl Zeiss, oberkohen, Germany) and Zeiss Sigma 300 scanning electron microscope (SEM) (FEI Company, Hillsboro, OR, USA) are used to observe the microstructure of microcapsules and coatings. The microscope needs to be adjusted to the appropriate multiple for observation.

(3) Infrared spectrum analysis: VERTEX 80V infrared spectrometer (FTIR) (Germany BRUKER Co., Ltd., Karlsruhe, Germany) is used to analyze chemical composition of the microcapsule and coatings, with a test range of 4000–500 cm^−1^ and a resolution of 4 cm^−1^.

#### 2.3.2. Performance Characterization of Antibacterial Coating Film

(1) Color difference: SEGT-J portable color difference instrument (Suzhou Weifu Photoelectric Technology Co., Ltd., Suzhou, China) is used to measure the color value of the water-based primer film with microcapsules. The calculation formula is as follows:

Δ*E* = [(ΔL*)^2^ + (Δa*)^2^ + (Δb*)^2^]^1/2^ (3)

The L* stands for brightness. The a* represents the red-green index of color. The b* stands for yellow-blue index. The chromaticity value of the film with and without microcapsules is tested.

(2) Gloss: LS195 intelligent glossmeter (Shenzhen Linshang Technology Co., Ltd., Shenzhen, China) is used to measure the gloss of coatings. It is mainly based on the glossiness at 60° incidence angle. The higher the brightness, the better the appearance quality of the coatings.

(3) Luminous transmittance of coating film: The coating on the glass substrate is used to measure the light transmission by U-3900 ultraviolet spectrophotometer (Shanghai Smeo Analytical Instrument Co., Ltd., Shanghai, China). The measurement range of transmittance value is 300–800 nm.

(4) Hardness: QHQ pencil hardness tester of 6H-6B (Shenzhen Forest Precision Instrument Co., Ltd., Guangzhou, China) is selected to measure the hardness of coatings. Pencils and pencils need to be tested at an included angle of 45° with a load of 1.0 kg. The hardness of the coating film scratched by a pencil is the coating film hardness.

(5) Adhesion: BYK cross-cut film scriber (Hebei Zhongke Beigong Test Instrument Co., Ltd., Cangzhou, China) is selected to test the film adhesion. The adhesion is divided into 6 levels, and the coating adhesion decreases from level 0 to level 5. The coating surface does not fall off when the grade is 0.

(6) Impact resistance: QCJ-120 film impactor (Shenzhen sanuo Experimental Equipment Co., Ltd., Shenzhen, China) is tested impact resistance. A 1.0 kg hammer falls on the coating from different heights to make the coating deform, and then the damage degree of the coating is checked. A 4× magnifying glass needs to be used to observe whether the coating has cracks and peeling. The maximum strength that does not cause damage to the paint film is denoted as impact resistance.

(7) Roughness: A precision roughness meter (Shanghai Taiming Optical Instrument Co., Ltd., Shanghai, China) is selected to measure the film roughness. The needle tracing or contact method measures one or several representative places on the main surface of the film. The roughness of the coating is comprehensively evaluated according to the measurement results. The unit of roughness is μm.

#### 2.3.3. Characterization of Antibacterial Property of Paint Film

Strain preservation: The strain of *Staphylococcus aureus* (inclined culture medium) is stored at 0–5 °C.

Activation of bacteria: The bacteria that have been stored for no more than 2 weeks are inoculated on the plate nutrient agar medium. They need to be cultured in a constant temperature and humidity chamber with a temperature of 36–38 °C. The required time is 18–20 h.

The inoculation ring scrapes the volume of fresh bacteria (1–2 rings) from the above two kinds of inoculated media. It is added to broth culture medium, and the selected concentration is (5.0–10.0) × 10^5^ cfu/mL (the total number of bacterial communities contained in each milliliter of sample).

Sample test: A total of 0.4 mL–0.5 mL of test bacterial solution is dripped onto the blank control template and antibacterial coating template, respectively.

Sterilized tweezers pick up the sterilized covering film and cover it on the sample plate, and then the film is pressed down slightly to spread the virus suspension around. It must be flat and free of bubbles, so that the bacteria can contact the sample evenly. The temperature of the constant temperature and humidity chamber is controlled at 38 °C, and the relative humidity is greater than 90% for 24 h.

Preparation of agar plate by dilution coating plate method: The sample and its covering film are taken out after 24 h of culture and put into sterilized beakers. Repeated use of 20 mL of eluent is needed to wash the paint film on the wood surface, and then the eluent is poured into the sterilized culture dish after fully shaking. The agar culture that was dissolved and cooled to about 45 °C is mixed with the eluent. After the agar solidifies, an agar plate that may contain bacteria is made. The colony can be produced after 24–48 h of culture at 36–37 °C. The results of the number of viable bacteria measured above need to be multiplied by 1000 for the actual number of viable bacteria recovered after 24 h of incubation of each sample. The unit is CFU/mL. The calculation formula is as follows:

R = (B − C)/B × 100% (4)

B = the number of colonies in the blank control sample without microcapsules;

C = the number of colonies in the sample with microcapsules.

All experimental errors were controlled within 5% and repeated 4 times.

## 3. Results and Discussion

### 3.1. Analysis of Orthogonal Experimental Results of UF @ Nanosilver Solution Microcapsules

#### 3.1.1. Analysis of the Output and Coverage Rate of Microcapsules

The mass ratio of the main antibacterial agent to the microcapsules is known as the coating rate. To assess the preparation of microcapsules, the coating rate of the core material is a crucial reference [39]. It is also the key to affect its antibacterial effect. The experimental findings of the orthogonal test are shown in Table 5. The variance analysis revealed that the emulsifier’s HLB value is the primary factor impacting the rate at which microcapsules encapsulate. According to the extremely poor results, the better preparation conditions are W_core_: W_wall_: 0.8:1, W_core_: W_emulsion_: 1:4, and the stirring speed is 1000 rpm/min.

It is also an important indicator in the preparation process to produce high-quality microcapsules with a small number of raw materials for its production and application [40]. The range and variance discovered by using the yield as the experimental outcome are displayed in Table 6 below. The variance results show that the emulsifier’s HLB value has the greatest impact on the creation of microcapsules. The better preparation process parameters are: W_core_: W_wall_ 0.8:1, W_core_: W_emulsion_ 1:4 and a stirring speed of 1000 r/min. The results of coating rate and yield show that the HLB value of emulsifier is the most influential factor.

**Figure 1:**
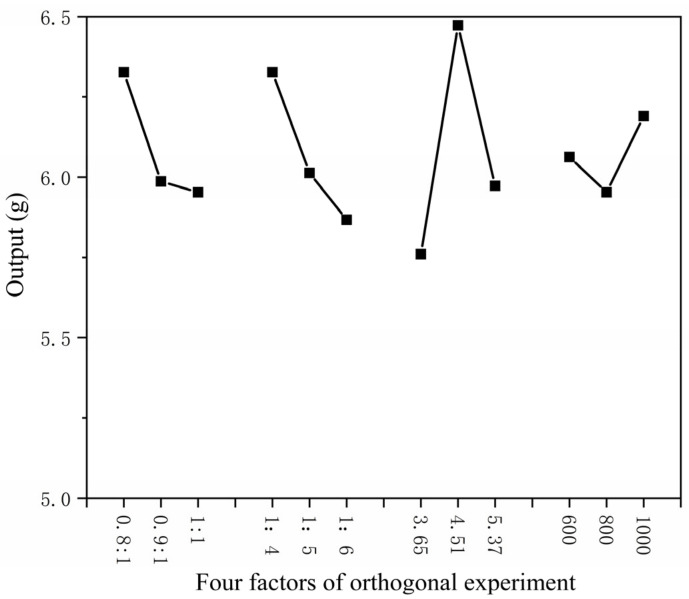
Relationship between four factors of orthogonal experiment and yield.

**Figure 2:**
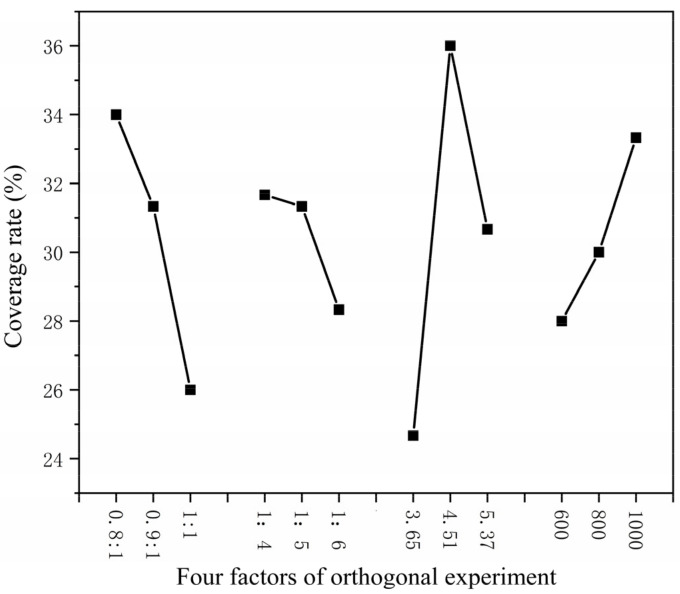
Relationship between four factors of orthogonal experiment coverage rate.

**Figure 3:**
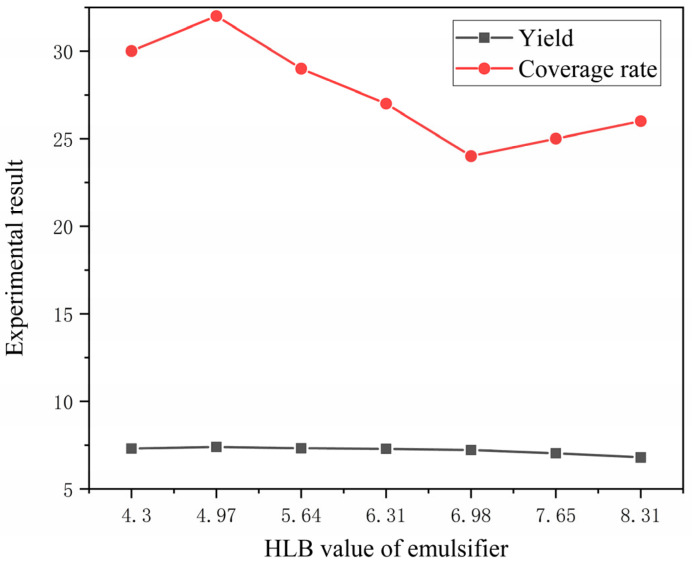
Relationship between HLB value and microcapsule yield and coverage rate.

The higher the core–wall ratio, the lower the output. The higher the mass ratio of core material and emulsifier in solvent, the lower the output as shown in Figure 1. The higher the HLB value of emulsifier, the lower the output. The faster the mixing speed, the higher the output. The coating rate has the same trend as the output, as shown in Figure 2. With the increase in emulsifier HLB value, the yield of microcapsules decreased, and the coating rate also showed a downward trend, as shown in Figure 3.

#### 3.1.2. Micromorphology Analysis of Microcapsules

**Figure 4:**
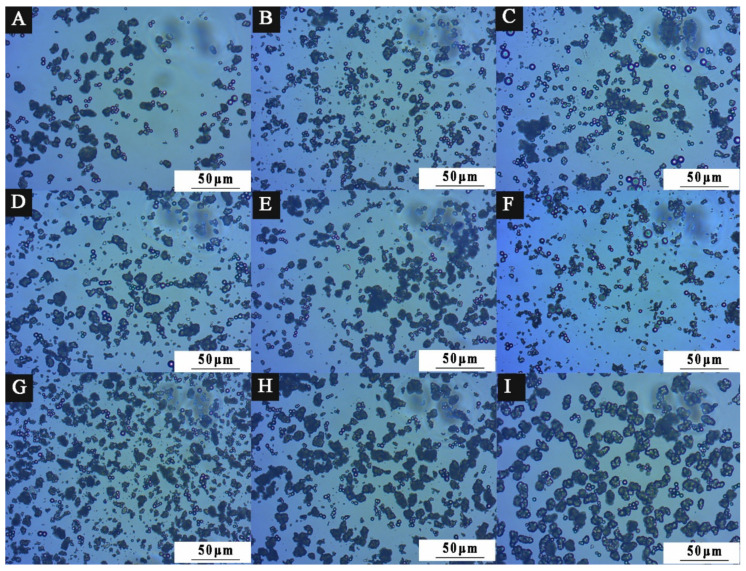
OM diagram of orthogonal experiment of 9 groups of microcapsules: (**A**) 1#, (**B**) 2#, (**C**) 3#, (**D**) 4#, (**E**) 5#, (**F**) 6#, (**G**) 7#, (**H**) 8#, (**I**) 9#.

Combined with the diffraction principle of light [41], the microcapsule powder presents a ring under the optical microscope, which is mainly produced at the interface of two different media. The white bright spot is the nanosilver solution core material. The dark ring is the wall material. It indicates that the microcapsule coating is successful. Figure 4 shows the OM diagram of nine groups of microcapsules designed by orthogonal experiment. Figure 4A shows the microcapsule morphology of 1# with an emulsifier HLB value of 4.3, Figure 4B shows the microcapsule morphology of 2# with an emulsifier HLB value of 4.97, and so on. Among them, 1#, 5#, 6#, 8#, and 9# microcapsules have obvious core–shell structure, in which the shape of the ball can be clearly seen, but there is a slight aggregation phenomenon. The distribution of 2#, 3#, 4#, and 7# microcapsules is relatively uniform. The microcapsules are not sticky to each other and have good dispersion. It shows that the ratio of emulsifier to core material and HLB value affect the stability of the emulsion solution of microcapsules, but a small number of spherical microcapsules can also be seen with obvious shell–core structure. The HLB value of the emulsifier affects the coating effect of the microcapsule. From the OM diagram, the smaller the HLB value of the emulsifier, the more obvious the spherical morphology, and the more microspheres. The higher the HLB value of the emulsifier, the easier it is for the microcapsules to agglomerate, making it difficult to see the microspheres. The result of this experiment is that with an emulsifier with an HLB value of 4.97, microcapsules with good morphology can be obtained.

### 3.2. Effect of Emulsifier HLB Value on Coating Film Properties

#### 3.2.1. Effect of the Emulsifier’s HLB Value on the Coating Film’s Optical Characteristics

**Figure 5:**
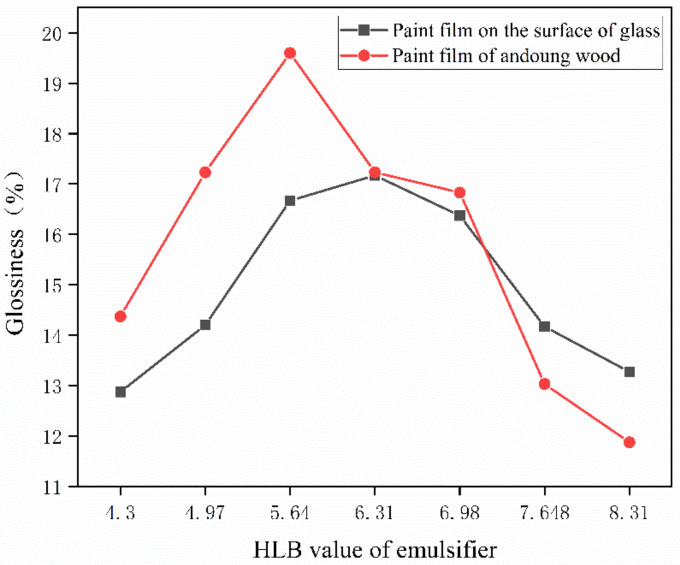
Effect of emulsifier HLB value on coating film glossiness.

One of the coating film’s most notable features, gloss, has a significant impact on the substrate’s ornamental qualities. Table 7 shows the glossiness values of samples of different substrates at an angle of incidence of 60° as a reference standard, where sample 0# represents the glossiness of the coated film without the addition of microcapsules. According to the test findings, the gloss of the film containing microcapsules is significantly lower than that of the film that does not include microcapsules. This may be because the microencapsulated powder is white, while the primer is transparent. There is a sense of particles when microcapsules are put to the primer, which causes the paint film’s surface to be uneven. The gloss of the coating film first increases and then decreases with the HLB value of the emulsifier, as shown in Figure 5.

**Figure 6:**
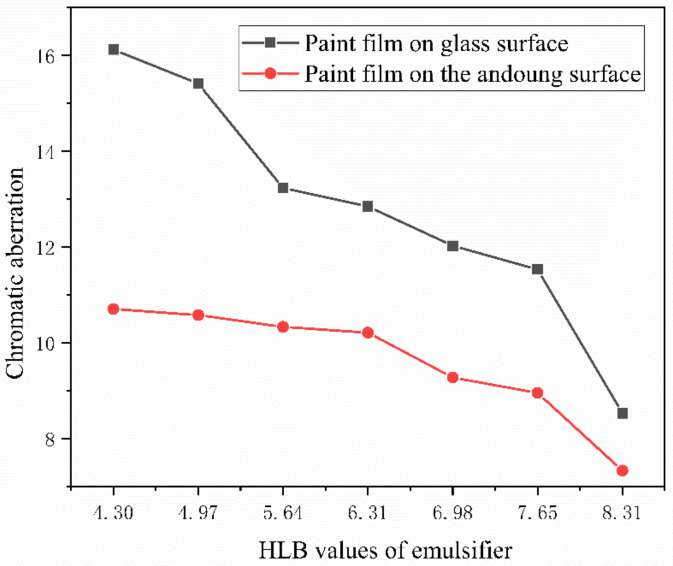
Effect of emulsifier HLB value on coating film color variation.

When light shines on the coating film, it absorbs, reflects, refracts, etc., which gives the coating film its color [42,43]. Color difference is a crucial characteristic to use when assessing the paint film’s optical qualities. The experiment’s blank control, a paint film devoid of microcapsules, had measured “*L*” values of 40.5, “*a*” value of 36.1, and “*b*” values of 29.5. The “*L*” value stands for the depth of the film color, the “*a*” value for the difference between red and green, and the “*b*” value for the difference between yellow and low-green. As can be seen in Figure 6, the color difference of the coated surface of the seven groups of samples clearly decreased as the HLB value increased. This demonstrates how the HLB value influences the coated film’s color variation. This might be due to the differences in color and transparency between nanosilver solution and Tween-80 and Span-80, which are transparent, thick, and yellow liquids, respectively. The mixed solution affects the overall color. The hue of the diffuse reflection of light is better than that of the wood surface coating due to the comparatively flat surface of the glass substrate.

**Figure 7:**
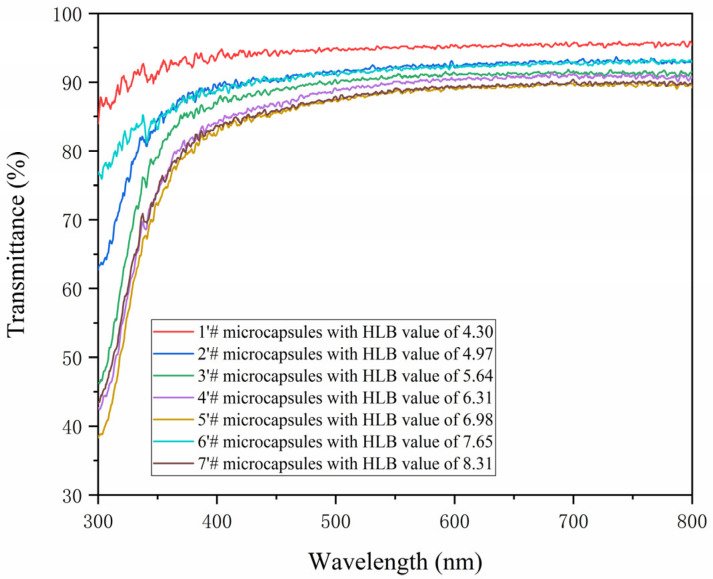
The transmittance diagram of the coating prepared by HLB values of different emulsifiers.

The light transmittance of the coating is a significant element influencing its optical characteristics [44]. Figure 7 depicts the measurement of the paint film’s light transmittance at a wavelength between 300 and 800 nm. At 380–780 nm (visible light transmittance), the transmittance of a coating film with 0# is approximately 91.7, whereas the coating film with 1′# has a transmittance of 94.9% and 2′# has a transmittance of 91.9%. It demonstrates that the use of antibacterial microcapsules in water-based coatings will increase the coating’s permeability while having no discernible impact on the paint film’s ability to transmit light. With the increase in the HLB value of the emulsifier, the transparency of the antibacterial coating prepared under visible light shows a downward trend, as shown in Table 8.

#### 3.2.2. Effect of Emulsifier HLB Value on Paint Film’s Mechanical Qualities

The analysis of the coating’s optical characteristics revealed that a coating with a high gloss level makes substrate flaws obvious. The requirements for the flatness and roughness uniformity of the substrate are relatively high [45,46]. As a protective coating, it is often subject to friction, impact, tension, and other forces, so the coating film is required to have necessary mechanical properties [47,48]. The mechanical characteristics of the coating film of the andoung wood surface are summarized in Table 9. The 0# sample without microcapsules was used as the control sample in the experiment, and its adhesion was good. With the addition of microcapsules with increasing HLB values into the coating, the adhesion began to decline in 4′# with an HLB value of about 7.64, which may be due to the destruction of the dense structure of the coating by microcapsules. This causes the coating’s mechanical qualities to deteriorate, which affects the coating film’s adherence to the surface of the wood. Adhesion describes how firmly the paint coating is adhered to the substrate. The paint coating is simple to pull off if the adherence is insufficient. However, the impact resistance clearly demonstrated a negative trend as the emulsifier’s HLB value increased, mainly due to the aggregation of microcapsules, which caused the film to crack when subjected to impact. However, the difference in hardness is small, which means that microcapsules have little effect on the coating film. The possible reason is that the coating film hardness is related to the composition and drying degree of the coating. Therefore, there is little difference in hardness. The film’s surface is flat and smooth when there are no microcapsules. However, when the microcapsules are added to the paint, the paint film’s surface gets rougher as the HLB value rises. The unequal distribution of the microcapsules in the paint film is a major contributor to the gaps in the paint film. The addition of nanosilver microcapsules reduces the surface adhesion and impact resistance of the paint film and has little impact on the hardness, as shown in Table 9.

### 3.3. Influence of the Emulsifier’s HLB Value on the Coated Film’s Antibacterial Properties

**Figure 8:**
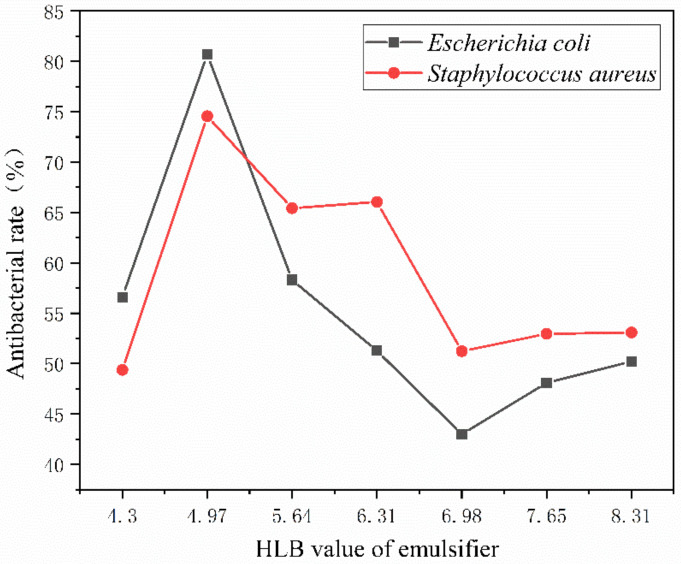
The antibacterial rate of coating film on glass substrate surface.

Figure 8 displays the antibacterial rate of antibacterial microcapsules created by emulsifiers with various HLB values and coated on glass using a water-based primer. *Escherichia coli* and *Staphylococcus aureus* were selected as the bacterial species for the blank control and antibacterial samples, respectively. The number of bacteria on the surface of glass plate without any treatment could not be counted. The number of viable bacteria on the surface of glass substrate without microcapsules reached 275 × 10^3^ cfu/mL. This shows that the water-based coating itself does not have the effect of inhibiting bacterial growth. As shown in the histogram, it can be seen intuitively that 2′# has the best antibacterial activity, with an antibacterial rate of 80.7% for *Escherichia coli* and 74.6% for *Staphylococcus aureus*. The antibacterial rate is first high and gradually decreases with an emulsifier’s rising HLB value. This shows that the lipophilic HLB value is around 6, making the preparation of nanosilver antibacterial microcapsules suitable. However, the higher the HLB value, the lower the antibacterial activity, as shown in Figure 8. The main reason for the low activity of *Escherichia coli* and the high activity of *Staphylococcus aureus* is the different activity of bacteria.

**Figure 9:**
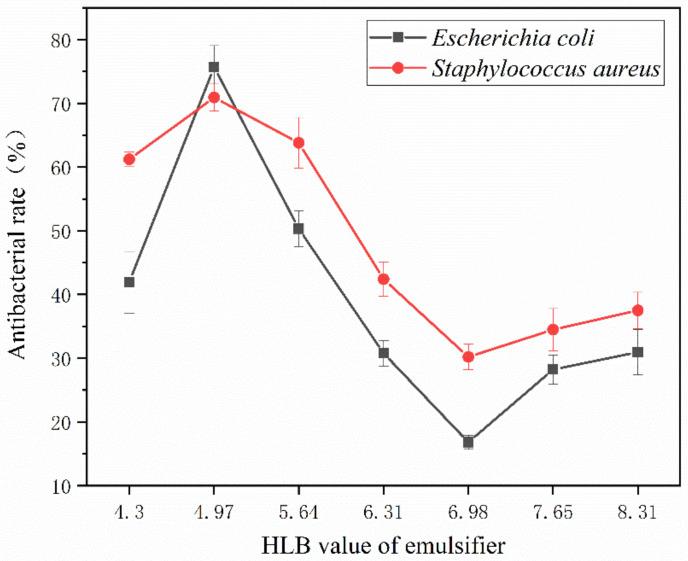
The antibacterial rate of coating film on the andoung wood surface.

Figure 9 shows the bactericidal rate of the coating film on the surface of the andoung wood. It is lower than the antibacterial rate of the glass substrate paint film, indicating that the wood surface is easily contaminated by bacteria. The highest antibacterial rate is 75.7% for 2′#, followed by 50.3% for 3′# and 16.8% for 5′#. It demonstrates that the antibacterial action, which primarily depends on the encapsulation of microcapsules, is the same on the surface of the wood substrate. It is made into microcapsules with urea-formaldehyde resin and applied to waterborne coatings to create an antibacterial layer. The nanosilver solution’s silver ion will be released when the microcapsule breaks upon contact with the bacteria.

When the amount of UF @ nanosilver solution microcapsules is 4.0%, the antibacterial rate of the coated film is 56.57%, which is much higher than the rate found in earlier studies. It mainly depends on the HLB of emulsifier, which ensures the stability of lotion, improves the encapsulation of microcapsule itself, and enables the nanosilver solution to be slowly released.

**Figure 10:**
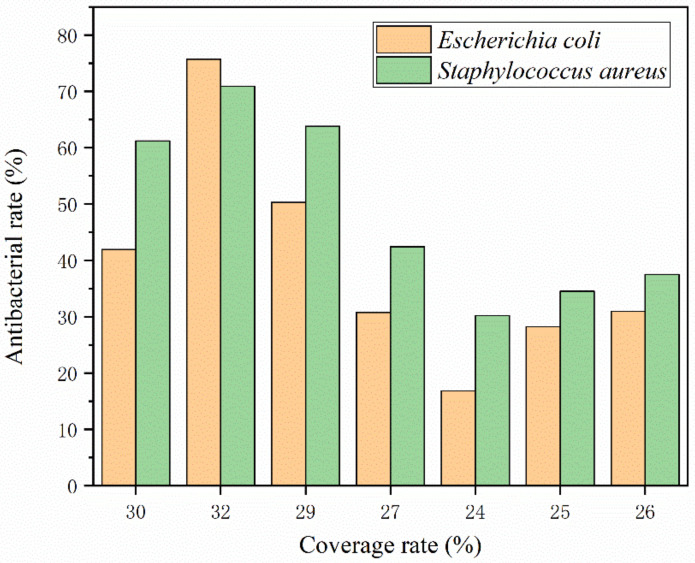
Relationship between coating rate and antibacterial rate.

The higher the coating rate of the nanosilver solution microcapsules, the better their antibacterial properties, as shown in Figure 10. The optimal lipophilic HLB value for preparing nanosilver antibacterial microcapsules is 4.97. The antibacterial component silver ion in nanosilver solution can destroy the cell membrane structure of bacteria and combine with protease to produce physical and chemical reactions to decompose protease. It can coagulate the protein in the bacteria and inactivate the bacteria to play an antibacterial role [49]. The core material nanosilver solution is coated in a urea-formaldehyde resin, and the surface of the wall material is of a microporous structure. The nanosilver solution flows out through the pores, releasing silver ions. Silver ions are positively charged and wood is not charged, and wood contains water, which can form OH-, forming electrostatic attraction and acting as an antibacterial agent with bacteria in the wood [50].

On the surface of the glass substrate, the antibacterial activity of the coating first rises and then decreases and tends to stabilize. The maximum antibacterial rate of the coating against *Escherichia coli* was 80.7%, and that against *Staphylococcus aureus* was 74.6%. On the surface of the andoung wood, the antibacterial rate of the coating increased first, with the highest antibacterial rate of 75.7%, followed by a downward trend in antibacterial rate. The antibacterial property of the wood surface coating is lower than that of the glass substrate surface, as shown in Table 10.

### 3.4. Analysis of the Micromorphology and Chemical Composition of Antibacterial Microcapsules with Varying Emulsifier HLB Values

**Figure 11:**
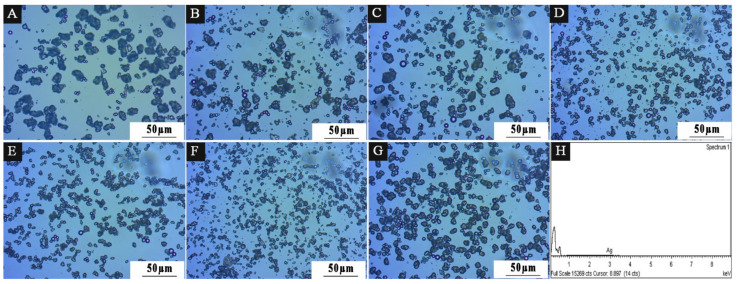
OM diagram of 7 groups of microcapsules in single-factor experiment: (**A**) 1′#, (**B**) 2′#, (**C**) 3′#, (**D**) 4′#, (**E**) 5′#, (**F**) 6′#, (**G**) 7′#, (**H**) EDS energy spectrum of 2′#.

Figure 11 displays the OM diagram for samples 1–7 in the experiment with a single factor. According to the diffraction ring phenomenon of light, white bright spots and black rings indicate a shell–core structure. Among them, the agglomeration of 2′# microcapsules is obviously better than that of other samples, and the agglomeration of 1′# microcapsules is the most serious. This shows that emulsifiers with different HLB values affect the preparation of microcapsules, mainly because emulsifiers play a key role in lotion polymerization. Anionic emulsifier Span-80 provides lotion with charge stability, which can prevent ion aggregation, and lotion has strong mechanical stability [51], but its chemical stability is poor. The nonionic emulsifier Tween-80 provides the lotion with spatial stability and good chemical stability [52].

**Figure 12:**
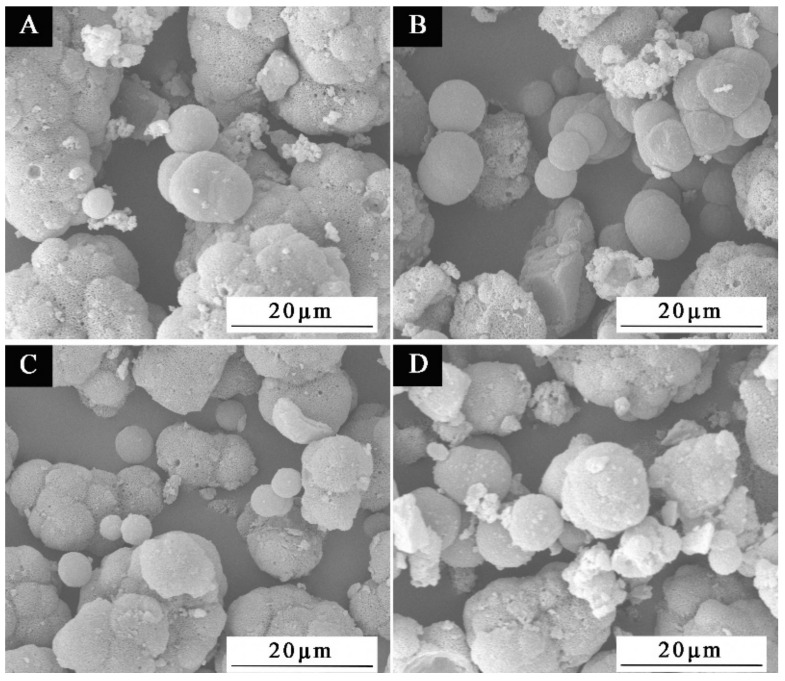
SEM images of microcapsules with HLB values of four emulsifiers: (**A**) 1′#, (**B**) 2′#, (**C**) 3′#, (**D**) 5′#.

SEM was used to examine the microscopic morphology of microcapsules in samples chosen for scanning electron microscopy based on the benefits and drawbacks of antibacterial rate and the size of HLB value in Figure 12. Sample 1′, which had the third-best antibacterial activity and lowest HLB value, along with samples 2′#, 3′#, and 5′#, which had the best, second-best antibacterial activity and biggest HLB value, were all chosen. The illustration makes it evident that the UF@ nanosilver solution microcapsules have a spherical shape. The 1′# sample in Figure 12A shows clearly that microcapsules are reunited. There are relatively more spherical microcapsules of 2′# sample in Figure 12B. In Figure 12C, the spherical microcapsules of sample 3′# are also present. The 5′# sample in Figure 12D shows some core material precipitation. The findings demonstrate that the encapsulation of microcapsules is impacted by the emulsifier’s high HLB value, which may be caused by the surfactant’s weak surface tension or interfacial tension. The adsorption on the interface after emulsification of core material is poor, resulting in poor emulsification and emulsion stability and dispersion. The smaller the HLB value of the emulsifier, the more obvious the spherical morphology and the more microspheres. The higher the HLB value of the emulsifier, the easier the microcapsules can agglomerate. The results of this experiment show that microcapsules with good morphology can be obtained by using the emulsifier with an HLB value of 4.97. The EDS spectrum of 2′# urea-formaldehyde resin-coated nanosilver solution microcapsules is shown in Figure 11H. It shows that the content of Ag in the microcapsules is very low. The reason is that only 0.02 mg of silver ions are contained in 1 g of nanosilver solution, so the characteristic peak appears weak.

**Figure 13:**
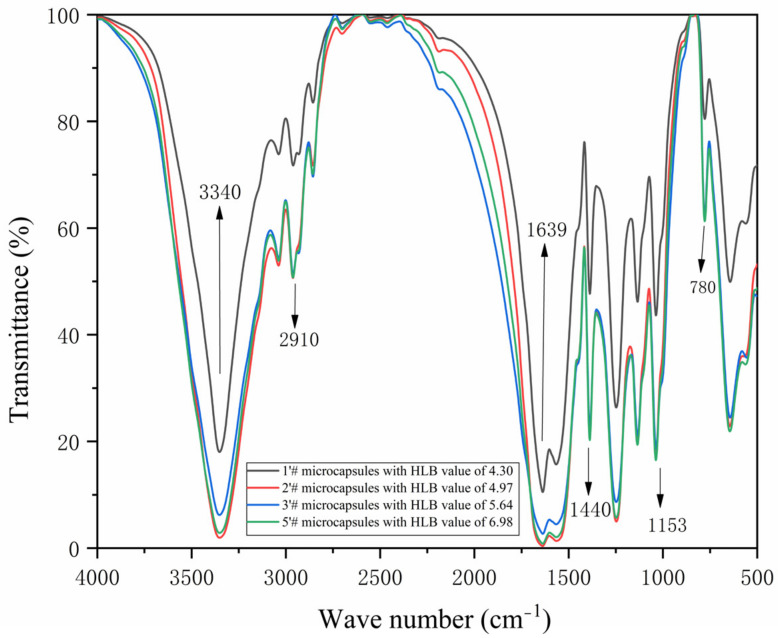
Infrared spectrum of microcapsules with HLB values of four emulsifiers: 1′#, 2′#, 3′#, 5′#.

The infrared spectra of the 1′#, 2′#, 3′#, and 5′# microcapsules are displayed in Figure 13. In nanosilver solution, it is the semiacetal C-O-C stretching vibration at 780 cm^−1^, while in urea-formaldehyde resin, it is the stretching vibration of C=O at 1639 cm^−1^ [53,54]. A superposition of -NH and -OH tensile vibration absorption occurs at 3340 cm^−1^. The telescopic vibration absorption of C-O-C is 1153 cm^−1^. Stretching vibration of -CH_2_ is at a peak of 1440 cm^−1^, while tensile vibration of CH_3_ is at a peak of 2910 cm^−1^ [55,56]. The above shows that the four microcapsules have been successfully coated. It is possible that emulsifiers with varying HLB values, which have an impact on the healing and film-forming response of water-based primer, are to blame for the wave peak fluctuation of paint film.

### 3.5. Analysis of the Coating’s Micromorphology and Chemical Composition before and after Antibiosis

**Figure 14:**
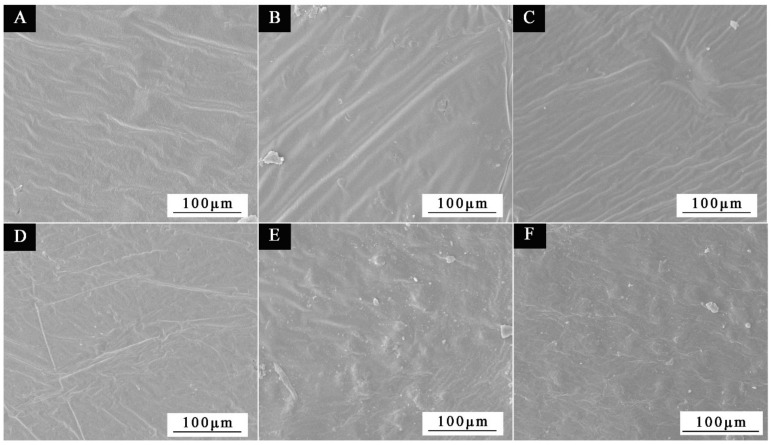
Coating film SEM pictures before antibiosis: (**A**) 0#, (**B**) 2′#, (**C**) 3′#; coating film SEM images before antibiosis: (**D**) 0#, (**E**) 2′#, (**F**) 3′#.

Before antibiosis, Figure 14A–C depicts the micromorphology of coating films with thicknesses of 0#, 2′#, and 3′#; after antibiosis, Figure 14D–F depicts the micromorphology of coating films on the andoung wood’s surface. The SEM picture reveals that the coating film’s surface is flat before antibiosis, but the paint film’s surface will have white spots after antibiosis. The cause is that the microcapsule’s breakage causes the core substance to leak out. Second, it is conceivable that bacteria on the coating film’s surface may eventually attach to and populate on the wood surface as a result of their contact with the material surface.

**Figure 15:**
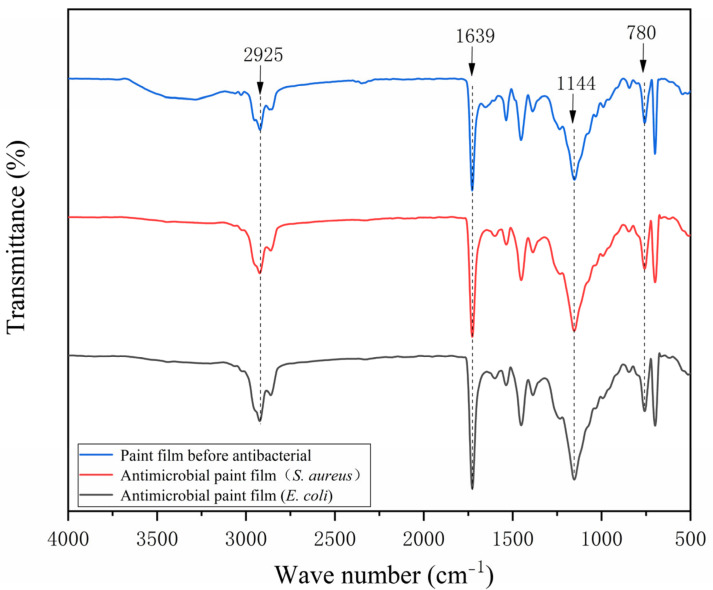
Infrared spectrum of 0# before and after antibacterial test.

**Figure 16:**
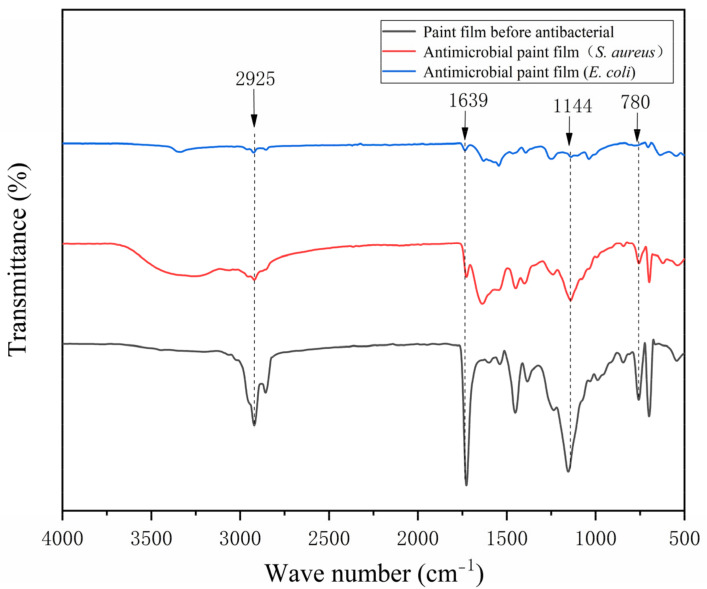
Infrared spectrum of 2′# before and after antibacterial test.

The FTIR spectrum of the pure-paint film before and after antibiosis without the addition of microcapsules is shown in Figure 15 and the FTIR spectrum of the 2′# optimum sample before and after antibiosis is shown in Figure 16. The urea-formaldehyde resin used as the primer’s microcapsule wall material has a telescopic vibration of C=O at 1639 cm^−1^ in it. The tensile vibration of -CH_3_ is measured at 925 cm^−1^, while the telescopic vibration absorption of C-O-C is measured at 1144 cm^−1^. The film with 0# microcapsules had no new peak before or after antibacterial treatment. In addition, the characteristic peak of 2′# before antibacterial treatment added with microcapsules is consistent with that of 0#, which indicates that microcapsules will not react with water-based primer. The characteristic peak of the 2′# coating with microcapsules was shortened after antibacterial treatment. The C-O-C in the nanosilver solution exhibits an asymmetric stretching vibration at 780 cm^−1^. It shows that there is no other product in the coating after antibacterial treatment, and the chemical composition has not changed. The rupture of the antibacterial microcapsule in the coating caused the outflow of the antibacterial nanosilver solution. Infrared analysis shows that silver ions are stable in the microcapsules without oxidation, so they can maintain antibacterial activity.

## 4. Conclusions

According to the results of the orthogonal experiment, the emulsifier’s HLB value has the greatest impact on the coating rate and yield. The nanosilver solution microcapsules coated with the urea-formaldehyde resin have good micromorphology by controlling the emulsifier’s HLB value. To create the antibacterial coating, the microcapsules were combined with 4% of the additive content in the water-based coating. This coating was then applied to the glass substrate to check the film’s antibacterial properties. According to the findings, microcapsules had an antibacterial activity of 74.55% against Staphylococcus aureus and an antibacterial activity of 80.7% against *Escherichia coli*. It demonstrates that the UF @ nanosilver solution microcapsules have antibacterial activity when combined with water-based primer. To investigate the effectiveness of the paint film, it was coated on the surface of the andoung wood. When the HLB value of the 2′# emulsifier was 4.97, the overall paint film performance was outstanding. The optical qualities of the film are impacted by the emulsifier’s HLB value. The best process preparation parameters are W_core_: W_wall_ is 0.8:1, W_core_: W_emulsifier_ is 1:4, HLB value of emulsifier is 4.97, and mixing speed is 1000 rpm. The film with the best antibacterial properties has a gloss of 14.37, a color difference of 10.58, and a transmittance of 93.1%. The hardness reaches 3H, the adhesion is level 2, and the impact resistance is 17 kg·cm, which has good mechanical properties. The antibacterial activity against *Escherichia coli* and *Staphylococcus aureus* reached 75.7% and 71.0%, respectively, while ensuring outstanding performance. The microcapsules did not react with the water-based coating, and there were no new chemical products before and after antibacterial treatment, according to chemical composition analysis, which may guarantee the microcapsule’s outstanding effectiveness. Through chemical composition analysis, no new products were produced before and after antibacterial treatment. This antibacterial microcapsule can be used for bacterial resistance on wood surfaces, even bamboo. The microcapsules can be added to the coating to achieve effective results, thereby expanding the application prospects of antibacterial microcapsules in coatings and other industries.

## Figures and Tables

**Table 1:** The list of the materials.

Materials	Specification/Size	Manufacturer
Urea	analytically pure	Tianjin Dengfeng Chemical Factory, Tianjin, China
37.0% formaldehyde	analytically pure	Nantong Yaoxin Chemical Co., Ltd., Nantong, China
Triethanolamine	analytically pure	Shaanxi Panlong Yihai Pharmaceutical Co., Ltd., Xi’an, China
Nanosilver solution	analytically pure	Tianjin Beichen Founder Reagent Factory, Tianjin, China
Span-80	analytically pure	Wuxi Yatai United Chemical Co., Ltd., Wuxi, China
Tween-80	analytically pure	Wuxi Yatai United Chemical Co., Ltd., Wuxi, China
Citric acid monohydrate	analytically pure	Nanjing Quanlong Biotechnology Co., Ltd., Nanjing, China
Absolute ethanol	analytically pure	Guangzhou Kema Chemical Technology Co., Ltd., Guangzhou, China
Waterborne paint	analytically pure	Akzo Nobel Paint Co., Ltd., Guangzhou, China
Escherichia coli	-	Beijing Conservation Biotechnology Co., Ltd., Beijing, China
Staphylococcus aureus	-	Beijing Conservation Biotechnology Co., Ltd., Beijing, China
Nutrient broth	-	Hangzhou Chicheng Pharmaceutical Technology Co., Ltd., Hangzhou, China
Eluent	-	Sichuan Kelun Pharmaceutical Co., Ltd., Chengdu, China
Nutrient agar medium	-	Guangdong huankai Microbial Technology Co., Ltd., Guangzhou, China

**Table 2:** Orthogonal test factors and levels.

Level	W_core_: W_wall_	HLB Value of Emulsifier	W_core_: W_emulsion_	Rotational Speed (rpm)
1	0.8:1	3.65	1:4	600
2	0.9:1	4.51	1:5	800
3	1:1	5.37	1:6	1000

**Table 3:** Orthogonal test schedule.

Sample (#)	Urea (g)	37% Formaldehyde (g)	Deionized Water (g)	Nanosilver Solution (g)	Span-80 (g)	Tween-80 (g)	Anhydrous Ethanol (g)	Rotational Speed (rpm)
1	7.0	9.5	35.0	8.4	0.5	0.1	33.0	600
2	7.0	9.5	35.0	8.4	0.7	0.1	41.2	800
3	7.0	9.5	35.0	8.4	0.9	0.1	49.4	1000
4	7.0	9.5	35.0	9.5	0.7	0.1	37.0	1000
5	7.0	9.5	35.0	9.5	0.9	0.1	49.3	600
6	7.0	9.5	35.0	9.5	0.5	0.1	56.1	800
7	7.0	9.5	35.0	10.5	0.9	0.1	41.0	800
8	7.0	9.5	35.0	10.5	0.5	0.1	51.9	1000
9	7.0	9.5	35.0	10.5	0.7	0.1	62.2	600

**Table 4:** The schedule for different HBL value.

Sample (#)	Urea (g)	37% Formaldehyde (g)	Deionized Water (g)	Nanosilver Solution (g)	Span-80 (g)	Tween-80 (g)	Anhydrous Ethanol (g)	Rotational Speed (rpm)
1′	7.0	9.5	35.0	8.4	4.3	0.8	0	33.6
2′	7.0	9.5	35.0	8.4	5.0	0.8	0.1	33.6
3′	7.0	9.5	35.0	8.4	5.6	0.7	0.1	33.6
4′	7.0	9.5	35.0	8.4	6.3	0.7	0.2	33.6
5′	7.0	9.5	35.0	8.4	7.0	0.6	0.2	33.6
6′	7.0	9.5	35.0	8.4	7.6	0.6	0.3	33.6
7′	7.0	9.5	35.0	8.4	8.3	0.5	0.3	33.6

**Table 5:** Orthogonal experiment results for coverage rate.

Sample	W_core_: W_wall_	W_core_: W_emulsion_	HLB Value of Emulsifier	Rotational Speed (r/min)	Coverage Rate (%)
1#	0.80:1	1:4	3.7	600	27.0
2#	0.80:1	1:5	4.5	800	40.0
3#	0.80:1	1:6	5.4	1000	35.0
4#	0.9:1	1:4	4.5	1000	41.0
5#	0.9:1	1:5	5.4	600	30.0
6#	0.9:1	1:6	3.7	800	23.0
7#	1:1	1:4	5.4	800	27.0
8#	1:1	1:5	3.7	1000	24.0
9#	1:1	1:6	4.5	600	27.0
Mean 1	34.0	31.7	24.7	28	
Mean 2	31.3	31.3	36.0	30	
Mean 3	26.0	28.3	30.7	33.3	
R	8.0	3.3	11.3	5.3	
Sum of Squared Deviations	99.6	20.2	192.9	43.6	
Degrees of Freedom	2	2	2	2	
F_ratio_	1.2	0.2	2.2	0.5	
F_critical_ Value	4.5	4.5	4.5	4.5	
Significance	-	-	-	-	

**Table 6:** Orthogonal experiment results for yield.

	Sample	W_core_: W_wall_	HLB Value of Emulsifier	W_core_: W_emulsion_	Rotational Speed (r/min)	Yield (g)
Range	1#	0.80:1	1:4	3.7	600	6.2
	2#	0.80:1	1:5	4.5	800	6.5
	3#	0.80:1	1:6	5.4	1000	6.1
	4#	0.9:1	1:4	4.5	1000	6.8
	5#	0.9:1	1:5	5.4	600	5.8
	6#	0.9:1	1:6	3.7	800	5.4
	7#	1:1	1:4	5.4	800	6.0
	8#	1:1	1:5	3.7	1000	5.7
	9#	1:1	1:6	4.5	600	6.2
	Mean 1	3.7	3.7	3.7	3.7	
	Mean 2	4.5	4.5	4.5	4.5	
	Mean 3	3.7	3.7	3.7	3.7	
	R	4.5	4.5	4.5	4.5	
Variance	Sum of Squared Deviations	0.2	0.3	420	18.7	
	Degrees of Freedom	2	2	2	2	
	F_ratio_	0.5	0.9	2.3	0.2	
	F_critical_ Value	4.5	4.5	4.5	4.5	
	Significance	-	-	-	-	

**Table 7:** The gloss of coating with different microcapsule content.

Sample (#)	Glass Substrate Surface Coating (%)	Andoung Wood Surface Coating (%)
0	67.00	45.37
1′	12.87	14.37
2′	14.20	17.23
3′	16.67	19.6
4′	17.17	17.23
5′	16.37	16.83
6′	14.17	13.03
7′	13.27	11.87

**Table 8:** Visible light transmittance of microcapsule antibacterial coatings with different HLB values.

Sample (#)	HLB Value of Emulsifier	Visible Light Transmittance (%)
0	0	91.7%
1′	4.30	94.9%
2′	4.97	91.9%
3′	5.64	90.2%
4′	6.31	89.1%
5′	6.98	87.8%
6′	7.65	91.6%
7′	8.31	88.1%

**Table 9:** Mechanical characteristics of the coating film applied to the andoung wood.

Sample (#)	Hardness (H)	Adhesion (Level)	Impact Strength (kg·cm)	Roughness (μm)
0	2	1	15	0.4
1′	1	2	15	1.2
2′	2	2	14	1.3
3′	2	2	14	1.8
4′	2	3	13	2.0
5′	1	3	13	2.4
6′	2	3	12	2.4
7′	1	3	11	2.5

**Table 10:** Antibacterial rate of Escherichia coli and Staphylococcus aureus on different substrates for waterborne primer films.

HLB Value of Emulsifier	Glass Substrate Surface		Andoung Wood Surface	
	*Escherichia coli* (%)	*Staphylococcus aureus* (%)	*Escherichia coli* (%)	*Staphylococcus aureus* (%)
4.30	56.6	49.4	41.9	61.2
4.97	80.7	74.6	75.7	70.9
5.64	58.3	65.4	50.3	63.8
6.31	51.3	66.0	30.8	42.4
6.98	43.0	51.2	16.8	30.2
7.65	48.1	53.0	28.2	34.5
8.31	50.2	53.1	31.0	37.5

## Data Availability

The data presented in this study are available on request from the corresponding author.
